# Post-Training Reward Partially Restores Chronic Stress Induced Effects in Mice

**DOI:** 10.1371/journal.pone.0039033

**Published:** 2012-06-22

**Authors:** Sergiu Dalm, E. Ron de Kloet, Melly S. Oitzl

**Affiliations:** Division of Medical Pharmacology, Leiden and Amsterdam Center for Drug Research, Leiden University Medical Center, Leiden University, Leiden, The Netherlands; Université Pierre et Marie Curie, France

## Abstract

Reduced responsiveness to positive stimuli is a core symptom of depression, known as anhedonia. In the present study, we assessed the expression of anhedonia in our chronic stress mouse model using a subset of read-out parameters. In line with this, we investigated in how far chronic stress would affect the facilitating effect of post-training self-administration of sugar, as we previously observed in naïve mice. Male C57BL/6J mice were repeatedly and at unpredictable times exposed to rats (no physical contact) over the course of two weeks. Following novelty exploration, (non-) spatial learning and memory processes with and without post-training sugar acting as reinforcer, emotionality, reward sensitivity and corticosterone levels were determined. We found that (1) the effects of chronic stress persisted beyond the period of the actual rat exposure. (2) Post-training self-administration of sugar as reinforcer improved spatial performance in naïve mice, whereas (3) in stressed mice sugar partially “normalized” the impaired performance to the level of controls without sugar. Chronic stress (4) increased behavioral inhibition in response to novelty; (5) induced dynamic changes in the pattern of circadian corticosterone secretion during the first week after rat stress and (6) increased the intake of sucrose and water. (7) Chronic stress and sugar consumed during spatial training facilitated the memory for the location of the sucrose bottle weeks later. Concluding, our chronic stress paradigm induces the expression of anhedonia in mice, at different levels of behavior. The behavioral inhibition appears to be long lasting in stressed mice. Interestingly, sugar consumed in close context with spatial learning partially rescued the stress-induced emotional and cognitive impairments. This suggests that reward can ameliorate part of the negative consequences of chronic stress on memory.

## Introduction

Chronic stress is considered a vulnerability factor for psychiatric disorders like depression [1], [2], [3]. One of the core symptoms of depression is anhedonia, i.e. the reduced reactivity to pleasurable stimuli or positive effects from events or activities that are normally rated as interesting or pleasant [4], [5], [6]. Anhedonia is considered to be the result of a disturbance in the detection of and response to positive emotional stimuli. The objective of the current study was to induce a disturbance in emotional processing by exposing mice to a chronic psychological stressor, and to investigate the reactivity to a rewarding stimulus. We measured emotional responsivity, cognitive performance and corticosterone secretion patterns.

Previous studies have shown that the repeated exposure of mice to rats, i.e., the ‘rat stress’ procedure, caused changes in the behavior of mice measured *during* and *directly after* ‘rat stress’ [7]. The behavioral changes included (i) inhibition of circadian activity patterns in the home cage, (ii) reduced sucrose consumption and inhibition of sucrose preference development and (iii) perseveration of behavior in a novel environment without a change in general locomotor activity. The same ‘rat stress’ protocol revealed changes in endocrine parameters together with impaired performance in hippocampus-dependent learning tasks [8], [9]. We also reported that chronic stress shifted the use of learning strategies towards favoring stimulus-response over hippocampus-dependent strategies in mice and man [10].

To assess whether our chronic stress procedure would induce the expression of anhedonia, we first determined several indicators for anhedonia. For this purpose we exploited the finding that positive stimuli and reward can strengthen memory traces [11], [12], [13]. In line with the theory of reward effects on memory we have demonstrated that post-training access to sugar facilitated spatial memory of mice in the water maze and the circular hole board task [14]. In the current study we studied the effect of post-training sugar on spatial performance in stressed mice, as an indicator for anhedonia.

Another indicator for anhedonia is derived from the consumption of and preference for a sweet solution. We and others have observed inhibition of consumption and preference for a sweet solution in close proximity to stress [7], [15], [16]. In contrast, long-term effects of stress and elevated glucocorticoids were reported to increase the consumption of and even preference for sweet solutions [17], [18]. Others have suggested that exploration patterns in a novel environment may provide leads to reveal the emotional state of the animal [19], [20]. Exploration is considered self-rewarding behavior. While the inhibition of exploration is generally related to anxiety, less exploration might also indicate the loss of hedonic responses, as suggested by Bevins and colleagues [5].

We examined the behavior of male C57BL/6J mice over the course of five weeks after cessation of the ‘rat stress’ procedure. During the first 4 weeks after stress, exploration patterns were determined in the novel environment of the circular hole board, in parallel with the measurement of spatial learning and memory performance and reversal learning, with and without post-training sugar as a reward. At 4 weeks after cessation of the ‘rat stress’ procedure, we measured the behavioral response to the light-dark box as an indicator for emotion-related behavior. Consumption and preference for a sucrose solution were assessed before, and 5 weeks after ‘rat stress’. To substantiate the paradigm of repeated rat exposure as a model for chronic stress, we measured circadian corticosterone secretion by taking blood samples three times a day, at one and six days after the last rat exposure.

We hypothesize that (i) chronic stress will impair spatial memory in mice and (ii) the memory facilitating effect of post-training sugar in stressed mice will be absent.

## Results

### Circular Hole Board: Novelty, Exploration and Search Strategies

**Figure 1:**
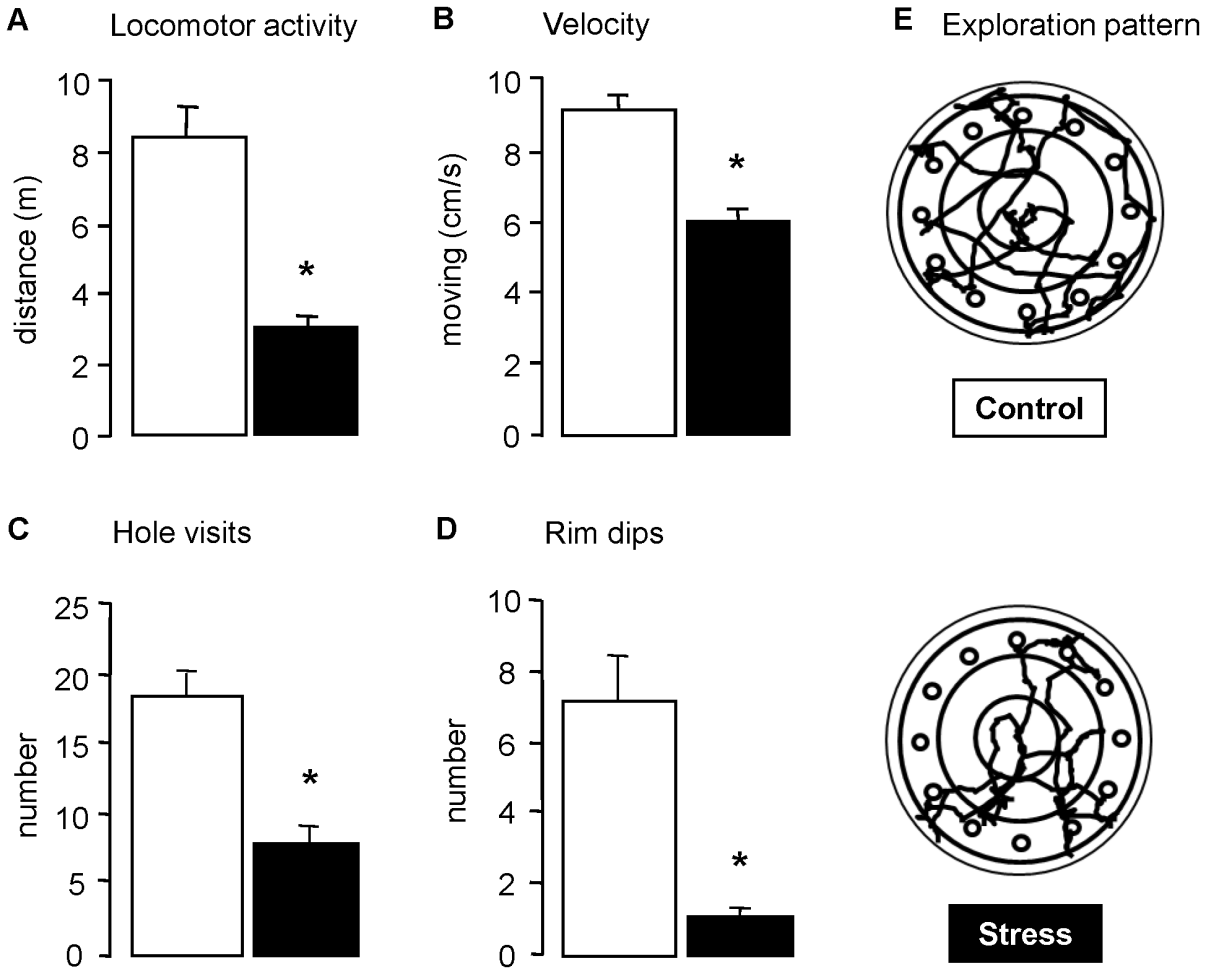
The experimental design of the study. Over the course of 9–10 weeks, male C57BL/6J mice were subjected to several procedures. The grey box highlights the time of the chronic stress procedure. Abbreviations: CHB  =  circular hole board; FET  =  free exploration trial.

One week after ‘rat stress’, we found a dramatically altered behavioral response of mice to novelty when exposed to the circular hole board (CHB), during the first free exploration trial (FET-1). Overall, behavior was suppressed in stressed mice, differing significantly between groups (F_(14,23)_ = 3.60, p = 0.001). General activity as expressed by path length in meters, velocity (cm/sec) and total number of hole visits (Figure 1A–C), was decreased (all p<0.01). Anxiety related behavior (all p<0.01), such as number of rim dips, was decreased (Figure 1D) while latency to the rim area was twice as long (stress: 205±25; control: 122±10). Behavior related to search strategies (all p<0.01) such as time (s) to leave the center (stress: 12.2±1.5; control: 6.5±1.1) and latency to first hole visit (stress: 21.7±4.7; control: 12.5±1.8) were increased in stressed mice. Most remarkably, stressed mice explored the CHB favouring the use of perseveration over serial strategy (%perseveration vs. %serial; stress: 69.1±7.3 vs. 31.3±9.7; control: 52.2±5.9 vs. 40.9±5.4; all p<0.01). An example of the walking pattern for a control and a stressed mouse can be seen in Figure 1E. Control and stressed mice were randomly assigned to sugar/no-sugar subgroups during spatial training on the CHB. These subgroups were comparable in their behavioral response to novelty (data not shown).

### Circular Hole Board: Spatial Training Trials 1 to 10

**Figure 2:**
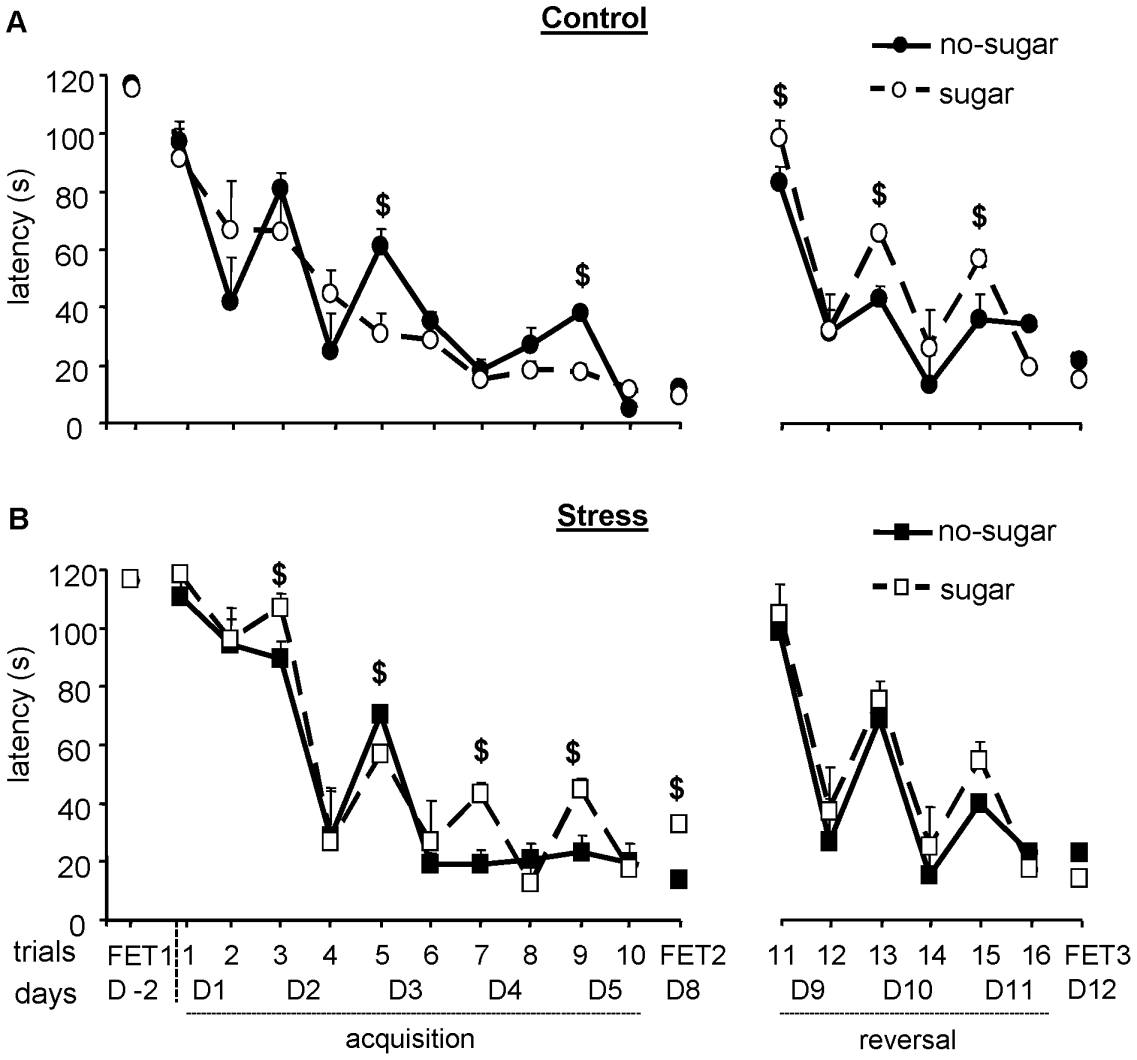
Chronic stress increases the behavioral inhibition of responses to novelty. Behavioral responses to the novel environment of the circular hole board (5 min free exploration trial - FET-1) were assessed one week after rat stress. A) Locomotor activity expressed as path length in meters; B) velocity on the board (cm/s); C) number of hole visits; D) number of rim dips; E) typical exploration pattern of a control and a stressed mouse. Data represent mean ± SEM; *p<0.05.

The learning curve, as expressed by the slope of latency and distance, decreased over trials (latency F_(4,72)_ = 54.67, p = 0.001; distance F_(4,72)_ = 6.08, p = 0.001); the pattern was different between control and stressed mice (trials*group: latency F_(11,396)_ = 3.15, p = 0.001; distance; p = 0.001). Stressed mice displayed a smoother learning curve vs. a seesaw-saw pattern for controls (Figure 2A). Walking velocity increased over trials (trials: F_(6,216)_ = 82.25, p = 0.001; data not shown). Path length was significantly shorter in stressed mice (trials*group: F_(11,396)_ = 5.03, p = 0.001; days 1, 2, 3, p<0.05; data not shown). The shorter path length during the first days was paralleled by a slower walking velocity in stressed mice (trials*group: F_(6,216)_ = 4.41, p = 0.001). On training day 1 and in the first trial of day 2, stressed mice took significantly longer to find the exit hole than controls (p<0.05; Figure 2AB).

Access to sugar after training resulted in a group-dependent effect on latency to reach the exit hole (Figure 2B). Control mice that received sugar showed a smoother learning curve than no-sugar controls. The latter had a typical seesaw pattern, where the latency for the first trial of the day was longer compared to the latency of the last trial of the previous day. Remarkably, stressed mice showed the opposite: with post-training sugar the pattern of performance was comparable to no-sugar controls; stressed mice without sugar showed a smooth learning curve. Post-training sugar did not affect the path length and the walking velocity to the exit hole in either group (trial*group*treatment: F_(11,396)_ = 1.13, p>0.05).

Over the course of the training trials, mice of both groups moved faster away from the start area (F_(6,216)_ = 69.25, p = 0.001; data not shown). However, stressed mice were significantly slower than controls to leave this area not only during FET-1 (before training p = 0.001), but also during training days 2, 3 and 5 (p<0.05) and FET-2 (after training; p = 0.003). Post-training sugar did not affect the time to leave the start area (time*group*treatment: F_(6,216)_ = 0.56, p>0.05).

### Circular Hole Board: Reversal Training Trials 11 to 16

During reversal training the exit hole had been relocated from position 3 to 11. The pattern of reversal learning resembles the original learning pattern (Figure 2AB): long latencies for the first trial, shorter latencies for the second trial of the day. Over days, mice of both groups learned the location of the new exit hole as indicated by a decrease in latencies over trials (F_(3,108)_ = 37.66, p = 0.001; path length F_(3,108)_ = 9.60, p = 0.001; data not shown). There was no main effect of stress on reversal learning. Control mice showed an effect of post-training sugar: controls with sugar displayed longer latencies in the first trial of the day (p<0.05). Walking velocity was group dependent (trial*group: F_(3,108)_ = 3.46, p = 0.019; data not shown) and significantly lower for stressed mice on days 10 and 11 (p<0.05). Time to leave the start area decreased group-dependently (trials*group: F_(3,108)_ = 3.70, p = 0.015; data not shown): stressed mice were significantly slower to leave the start area than controls. Interestingly, post-training sugar had group-dependent effects on this parameter (group*treatment: F_(3,108)_ = 6.18, p = 0.018). Control mice with sugar were significantly slower to leave the start area than controls in the first trial on days 9, 10 and 11 (p<0.05); also their latencies to the exit hole are longer. Stressed mice with sugar were faster to leave the start area than stressed without sugar on day 9 (p = 0.041), however, the latencies to the exit hole are the same in both groups.

### Behavior during Free Exploration Trials After Training

During FET-2 and FET-3 all holes are closed. In comparison to the behavioral response during FET-1 before training, general activity of controls and stressed mice was increased, i.e., path length, speed of moving, and total hole visits (Table 1). Goal-directed behavior became more prominent. The search strategy shifted from perseveration to serial, the latency to the previous learning exit hole decreased, and controls and stressed mice visited the exit hole more often.

**Table 1:** Behavioral parameters determined during the 5 min free exploration trials (FET-2 and FET-3).

	FET–2 after spatial acquisition				FET-3 after reversal training			
Behavioral parameters	Control		Stress		Control		Stress	
*General activity*	*no-sugar*	*sugar*	*no-sugar*	*sugar*	*no-sugar*	*sugar*	*no-sugar*	*sugar*
Path length (m)	15.9±0.6	15.6±0.9	14.8±0.9	**15.0±1.3^∼^**	13.8±1.1	15.9±0.9	15.4±2.0	18.7±1.1
Speed of moving (cm/s)	13.8±0.3	13.0±0.6	12.8±0.8	**12.1±0.5^∼^**	13.5±0.3	13.1±0.5	13.6±0.8	14.8±0.4
Total hole visits	54.1±2.1	53.1±2.9	52.2±5.8	58.2±3.2	48.6±5.1	50.1±1.8	**48.1±4.6** #	63.9±4.0
***Search strategy***								
Latency (s) from start center	1.5±0.2	**1.7±0.3^∼^**	**3.0±0.5** *	**3.2±0.5** *	2.5±0.5	3.4±1.0	3.7±0.6	3.2±0.6
Latency (s) 1ste hole visit	**2.6±0.3** #	4.7±0.6	4.0±0.6	4.4±0.7	**3.0±0.4** #	6.1±0.7	5.0±0.7	3.5±0.3
Latency (s) 1^st^ hole dip	5.0±0.3	**8.0±0.6^∼^**	**10.8±2.4** *	**11.1±1.5** *	**6.0±1.0** #	13.7±2.2	10.0±1.6	8.3±1.3
Latency (s) exit hole 3	**12.3±1.3^∼^**	**9.3±0.9^∼^**	**13.6±2.4** # * **^∼^**	**32.9±9.0** *	27.4±3.4	46.5±13.2	**62.4±17.3** #	20.6±4.2
Number of visits exit hole 3	**8.6±0.7^∼^**	**6.5±0.5^∼^**	**9.4±1.4^∼^**	**8.8±0.7^∼^**	4.1±0.6	3.5±0.3	**5.1±0.7** *	**6.7±0.7** *
Latency(s) exit hole 11	n.a.	n.a.	n.a.	n.a.	21.7±7.1	26.8±4.7	23.0±4.6	14.0±2.8
Number of visits exit hole 11	**3.3±0.3^∼^**	**3.0±0.3^∼^**	**2.5±0.8^∼^**	**2.6±0.3^∼^**	7.1±0.5	5.4±0.6	5.3±0.7	6.9±0.9
% Serial	76.7±3.7	80.0±3.9	75.9±4.9	80.8±3.5	81.0±4.9	88.9±2.6	77.9±4.4	85.7±3.3
% Perseveration	39.8±2.3	36.3±3.8	7.1±4.9	**43.4±4.0^∼^**	34.2±3.1	35.8±3.1	**38.5±3.9** #	27.7±2.7
***Anxiety-related***								
Latency (s) to rim	**88.1±19.7^∼^**	118.8±17.8	**128.9±7.8** *	**165.4±21.8** * **^∼^**	**212.3±30.5** #	126.1±18.0	**119.6±11.8** # *	**58.5±12.6** *
Number of rim dips	**12.0±2.2^#∼^**	7.2±0.9	**8.0±1.4** #	**3.0±0.4^∼^**	5.3±0.8	6.1±1.0	**11.1±1.3** *	**11.9±1.3** *

FET-2 was assessed three days after spatial training, and FET-3 one day after reversal training. Data represent mean ± SEM. Behavioral parameters that differ significantly are **bold**; p<0.05.

* between groups control vs. stress;

# within groups, **^∼^**FET-2 vs. FET-3; n.a.  =  not applicable.

Spatial acquisition training differentially affected the behavioral response of control and stressed mice as observed during FET-2 (MANOVA: F_(14, 23)_ = 4.54, p = 0.001). Stressed mice were slower than controls to leave the start area and to locate the exit hole. Controls with sugar had less rim dips and visits to the exit hole, yet, were faster in locating the exit tunnel than no-sugar controls. Similarly, stressed mice with sugar had less rim dips than stressed no-sugar mice, while their number of visits to the exit hole was unaffected. The latency to the exit hole of stressed mice with sugar was twice as long as in the stressed without sugar mice.

The FET-3 following reversal learning revealed group differences in the behavioral response (MANOVA; F_(14,23)_ = 2.11, p = 0.05). Stressed mice made more rim dips than controls, while general activity was similar between groups. Sugar had no effect in the control group. However, stressed mice with sugar had a significantly longer path length, faster walking velocity and more hole visits than stressed no-sugar mice (all p<0.05). Furthermore, stressed mice with sugar reached the rim of the board faster and made more rim dips. The search strategy employed was similar between groups. Perseveration was less expressed in stressed mice with sugar than stressed mice without sugar.

Interestingly, memory related parameters differed according to group and treatment. Control mice visited the “new” exit (from the reversal training) about twice as much than the “old” exit (from the initial training); stressed mice visited the “new” and “old” location comparably often (all p<0.05). It took stressed mice with sugar one third of the time to locate the “old” exit hole compared to stressed mice without sugar (group*treatment: F_(1,36)_ = 7.37, p = 0.023). Also latencies to “new” and “old” exits were shortest in stressed mice with sugar.

### Persistence of Directed Search Following Spatial Training

**Figure 3:**
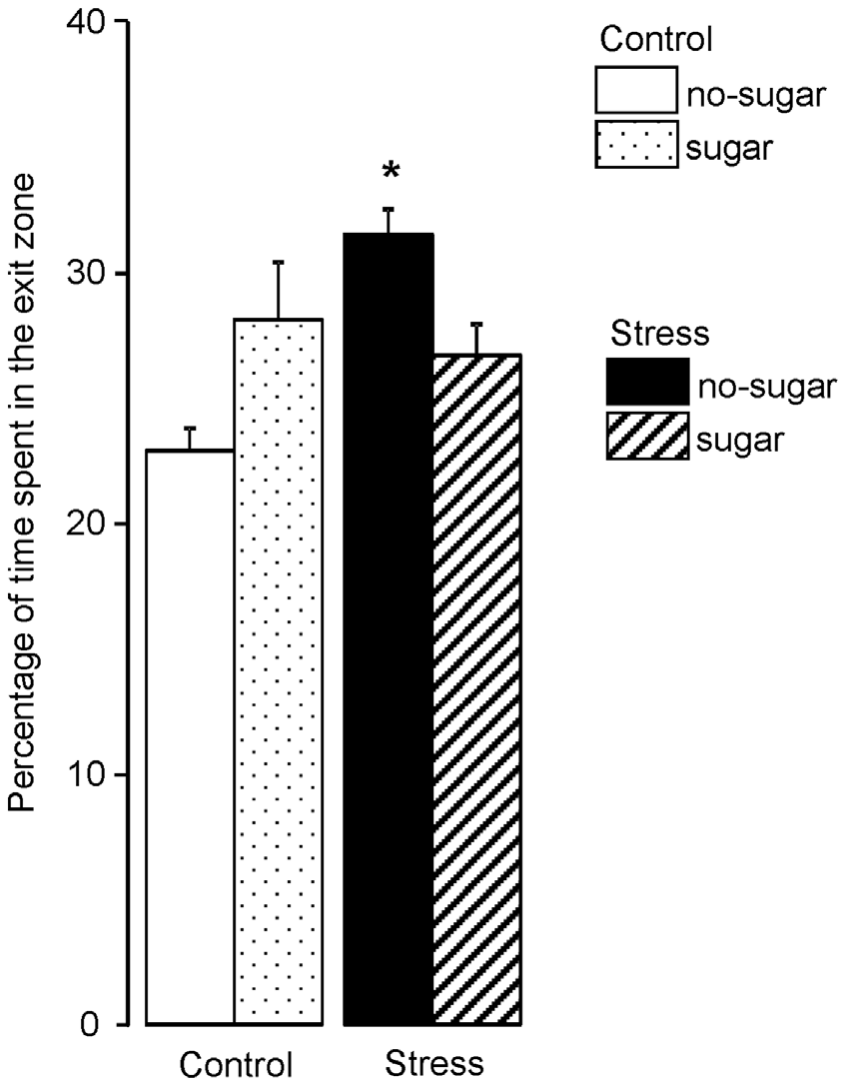
Chronic stress alters spatial performance, whereas post-training sugar partially restores performance. Three days before spatial training started (D-2) a free exploration trial (FET1) was conducted. Spatial performance on the circular hole board (mean of 2 trials/day) expressed as A) latency in seconds and B) path length in meters to the exit hole during the 1 spatial acquisition (training days D1–5) and reversal (training days D9–11). A subgroup of control and stressed mice had daily free access to sugar (30 mg/day) in their home cage after the last training trial. For FET2 and FET3, the latency and distance moved relate to the first exit hole visit. Data represent mean ± SEM; *p<0.05 between groups.

During 5 days of spatial training mice learned to locate the exit hole. The persistence of search was defined by the percentage time spent in the area at the location of the previously accessible exit hole (15 cm radius), during the 5 min of FET-2 (Figure 3). Stress and sugar affected the time spent close to the exit hole. Stressed mice remained longer in the exit area than controls (main effect of group F_(1,36)_ = 5.94, p = 0.020). The effect of sugar on control and stressed mice was opposite (group*treatment F_(1,36)_ = 11.30, p = 0.002): sugar during training increased the time in the exit area in control mice (p = 0.018) whilst decreasing it in stressed mice (p = 0.029). Consequently, the persistence behavior of control mice with sugar was statistically comparable to stressed mice that had received sugar during training.

### Behavior in the Light-dark Box

Four weeks after the last rat exposure, mice were placed in the light compartment of the light-dark box, and tested for light-dark preference. Stressed and control mice responded differently (Table 2: MANOVA: group: F_(5,32)_ = 5.17, p = 0.001). Stressed mice took more time to enter the dark compartment (F_(1,36)_ = 12.30, p = 0.001), spent more time in the light compartment (F_(1,36)_ = 16.58, p = 0.001) and had a longer path length (F_(1,36)_ = 11.04, p = 0.002) than controls. Walking velocity in the light compartment was comparable between groups.

**Table 2:** Behavioral parameters expressed in the light area of the light-dark box during 5 min exposure.

	Control		Stress	
Behavioral parameters	*no-sugar*	*sugar*	*no-sugar*	*sugar*
Latency (s) to dark*	7.6±1.3	6.6±0.6	13.1±1.4	**9.7±1.4** #
Latency (s) to light	34.1±4.2	**21.6±1.9** #	29.3±2.4	28.9±3.4
Path length (m)	5.0±0.3	5.0±0.6	6.8±1.3	6.2±0.6
% Time spent*	25.7±1.5	30.1±2.7	42.2±2.2	**32.2±2.8** #
Speed of moving (cm/s)	6.2±0.2	6.2±0.2	5.4±0.3	**6.5±0.3** #

Data represent mean ± S.E.M. Behavioral parameters that differ significantly are **bold**; p<0.05.

* between groups control vs. stress;

# within groups.

Sugar had distinct effects on behavior of controls and stressed mice (group*treatment: F_(5,32)_ = 3.49, p = 0.013). Stressed mice with sugar had shorter latencies to the dark compartment and spent less time in the light compartment and their walking velocity was higher than in stressed mice without sugar (all p<0.01). Control mice with sugar were faster to re-enter, and spent more time in the light compartment than controls without sugar (both p<0.05); walking velocity was comparable.

### Sucrose Consumption and Preference

Control and stressed mice preferred sucrose solution over water. We calculated the difference in fluid intake (5% sucrose-, water- and total fluid consumption in ml) between baseline (day 14; i.e., 4 days before the rat stress paradigm started) and 5 weeks after the last rat exposure (day 63, Table 3). Stressed mice drank more of the sucrose solution and water than controls (group: sucrose F_(1,36)_ = 9.02, p = 0.005; water F_(1,36)_ = 4.71, p = 0.037), with a significantly higher total fluid consumption (p = 0.002). Sugar during CHB training had no effect on fluid consumption of controls and stressed mice.

**Table 3:** The consumption (ml) of and preference (%) for drinking a 5% sucrose solution and water during 24 h.

	Consumption (ml)			Preference (%)			
	(day 14 baseline vs. day 63)			day 63		day 64	
Group	sucrose	water	total	sucrose	water	water	water
Control, no-sugar	−1.6±1.3	0.6±0.2	−1.0±1.2	88.0±0.6	11.5±0.8	45.9±1.8	54.1±1.8
Control, sugar	−0.3±1.0	0.8±0.1	0.5±1.0	88.1±0.5	11.9±0.5	50.3±1.7	49.7±1.7
Stress, no-sugar	2.3±0.5	0.9±0.1	1.0±0.1	86.8±0.8	13.2±0.8	52.3±4.7	47.7±2.7
Stress, sugar	1.4±0.6	3.3±0.6	2.5±0.6	87.7±0.6	12.3±0.6	**62.6±2.2** $	37.4±1.2

On day 63 one bottle contained sucrose, the other contained water. On day 64, both bottles contained water. Data represent mean ± SEM. Behavioral parameters that differ significantly are **bold**; p<0.05.

* between groups controls vs. stress;

$ vs. all other groups.

Immediately following the 24 h sucrose consumption test on day 63, the sucrose bottle was replaced by a water bottle. Water intake from both bottles was determined 24 h later (day 64–65). The total water intake was similar, in the range of 10 ml in all groups. However, stressed mice drank more from the water bottle which had replaced the sucrose bottle (group: F_(1,36)_ = 18.92, p = 0.001). This is due to the stressed mice that had received sugar during CHB training. These mice had the highest preference for the water bottle at the location of the previous sucrose bottle (stress with sugar vs. all groups F_(3,39)_ = 10.85, p = 0.002).

#### Effects of chronic rat stress on circadian corticosterone secretion

**Figure 4:**
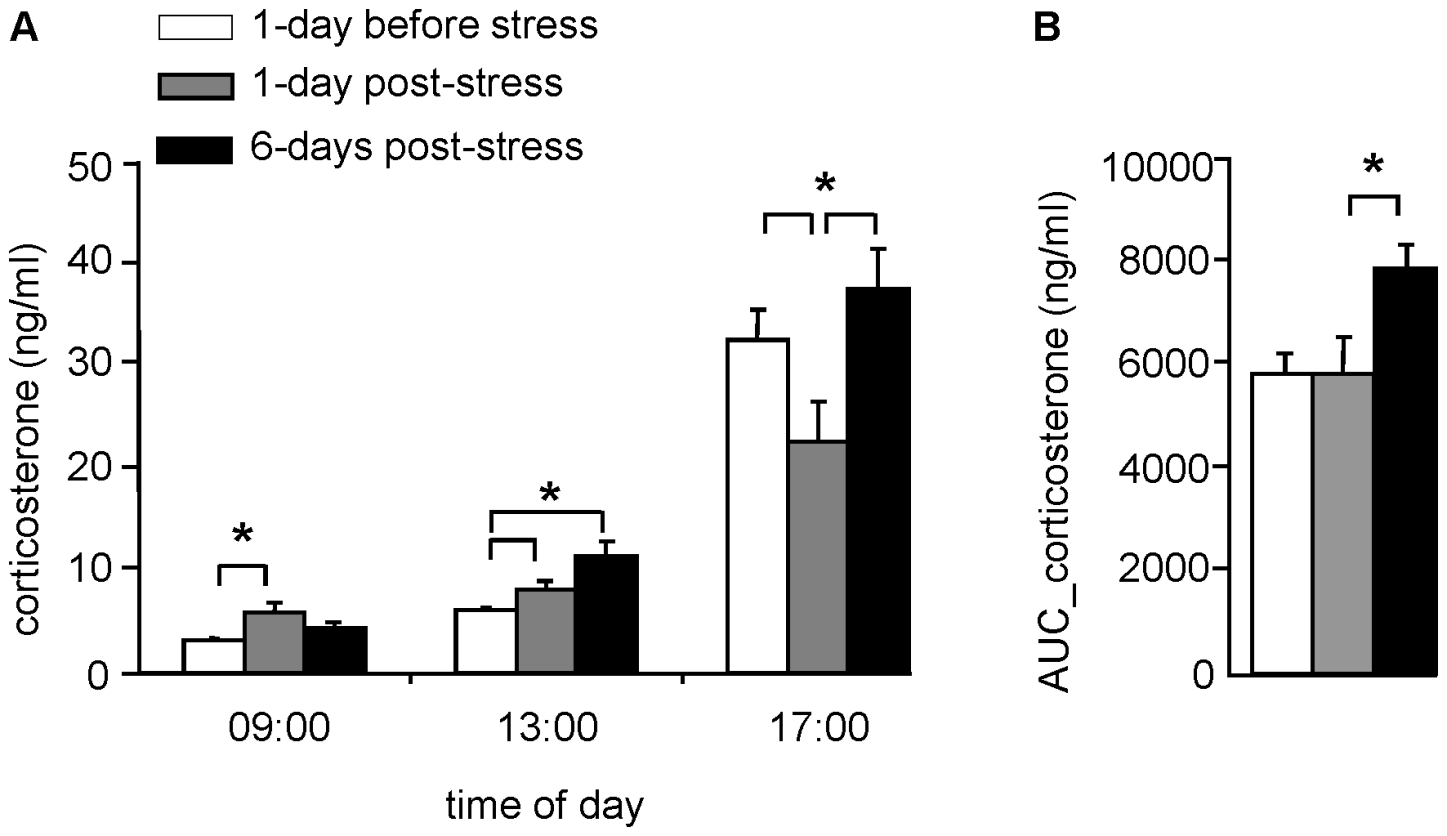
Stressed mice display reduced flexibility in directed search; sugar partially restores. Three days after the last spatial acquisition training trial, the percentage of time spent in the exit zone (15 cm radius) was determined during 5 min of free exploration trial 2 (FET-2). Data represent mean ± SEM; p<0.05 *control vs. stress; ^∼^no-sugar vs. sugar.

Rat stress changed the pattern of corticosterone secretion differentially, depending on the post-stress day of measurement (Figure 4A; time*group F_(4,114)_ = 4.53; p = 0.002). Corticosterone secretion increased over the day (time effect: F_(2,114)_ = 246.26; p = 0.001). One day post-stress, corticosterone concentrations were higher at 09∶00 a.m. and 13∶00 p.m. compared to the before-stress condition (p = 0.001), but lower at 17∶00 p.m. compared to before-stress and 6-days-post-stress conditions (p<0.05). Remarkably, 6-days post-stress, the overall circadian corticosterone surge during the light period was augmented (Figure 4B: Area_Under_Curve: one-way ANOVA F_(2,59)_ = 7.52, p = 0.020). In contrast, overall corticosterone concentration during the light period was similar between before-stress and 1-day-post-stress conditions (p>0.05).

#### Body weight

All mice gained weight over the course of the experiment (about 13%; day 1: controls 24.7±0.2; mice that will be stressed 24.5±0.2; end of experiment controls 27.2±0.2; stressed 28.2±0.3). Chronic stress did not affect body weight.

## Discussion

The phenotype of the chronically stressed mice has a strong resemblance with features of depression in humans. The effects of the chronic ‘rat stress’ model persisted beyond the period of actual exposure to the rat. One to five weeks after cessation of the stressor, we observed suppression of behavioral reactivity together with altered spatial learning and memory and emotionality. In addition, the pattern of circadian corticosterone secretion showed dynamic changes during the first week after rat stress, culminating in an overall increase in total corticosterone exposure during the light period of day 6. Reward sensitivity was affected as indicated by distinct sensitivity of memory to sugar reward: spatial performance improved in control mice whereas in stressed mice sugar reward “normalized” performance to the level of controls without sugar. Also, an increased sucrose and water intake in stressed mice and preference to drink water at the location of prior sucrose consumption was observed. Remarkably, sugar consumption in close context with spatial learning partially rescued stress-induced emotional and cognitive disturbances, with the effects measured even weeks later in other tasks. Although the increase in sucrose consumption and a similar preference compared to non-stressed mice are not characteristic for anhedonia, they do reflect an alteration in the reward system.

### Chronic Stress and the Expression of Anhedonia

We used a variety of parameters that indicate emotional and cognitive responses in relation to positive stimuli that could be affected by chronic stress: approach behavior, post-training sugar administration and sucrose-preference testing.

### Behavioral Inhibition

Exploration of novel environments is an essential aspect of behavior. At the same time, the exposure to novelty creates a conflict between approach towards new sources of reward and avoidance of potential treats [21], [22]. Previously, [7] we exposed chronically stressed mice to the circular hole board two days after the last stressor. Behavioral changes were limited to reduced latency to first hole visit and increased perseveration. In the present study, chronically stressed mice displayed strong behavioral inhibition upon exposure to the novel environment of the circular hole board, one week after cessation of the stressor. The inhibition remained even during recurring training and free exploration trials on the circular hole board, i.e. stressed mice were always slower to leave the start area of the circular hole board. However, over trials the latency to locate the exit hole decreased to the level of non-stressed mice, indicating the learning capability of stressed mice. Interestingly, 5 weeks after the last rat exposure, stressed mice still displayed behavioral inhibition when exposed to the novel environment of the light-dark box. We previously observed a similar response to the light-dark box for stressed mice, even 3 months after cessation of the stressor [9]. We may conclude that chronic stress has long-lasting consequences as expressed in different degrees of behavioral inhibition in novel environments.

Approach behavior may yield important information about food and reproduction possibilities, while an exposed illuminated place, for example, is dangerous with regard to predators and has to be avoided. Indeed, non-stressed mice explored the novel environment of the circular hole board, while also moving away from the brightly lit open space during light-dark box testing. Stressed mice lack the anticipatory responses: their behavior is inhibited and non-adaptive on both the circular hole board and the light-dark box. Chronic stress also reduced the activity of mice in the familiar environment of the home cage [7]. In that study, we showed that the activity was dedicated to foraging (moving to and from the food dispenser) at the expense of moving around in other areas of the cage. It is evident that chronic stress resulted in a shift of approach/avoidance behavior and thus, a lack of behavioral adaptation in novel environments. Bevins and Besheer [5] interpreted such results as changes in reward sensitivity. Therefore, the behavioral inhibition in stressed mice might point towards an alteration in reward that will influence memory formation.

### Modulation of Learning and Memory by Post-training Reward

Chronic stress and long term exposure to high levels of glucocorticoids are known to alter neuronal morphology and synaptic plasticity in the hippocampus (spatial memory for facts), prefrontal cortex (response selection), striatum (stimulus-response) and amygdala (emotional value of stimuli), amongst other structures, affecting spatial processing [23], [24], [25], [26], [27], [28]. Reward-coding dopaminergic neurons in the hippocampus regulate the motivational drive to explore an environment. They are involved in signaling stimulus novelty and are able to facilitate hippocampus-dependent consolidation memory of novel events [29]. We had hypothesized that the impact of chronic stress on the modulation of memory by post-training administration of sugar would indicate a change in the reward system of the mice. Post-training reward has been shown to strengthen memory traces [11], [12], [13]. Recently, we demonstrated that access to sugar directly post-training resulted in the improved spatial memory of mice in a water maze and circular hole board task [14].

We will discuss the impact of chronic stress followed by the effects of post-training sugar on learning and memory processes. Chronic stress impaired learning which is in accordance with the literature [25] and our own previous findings on the circular hole board task using an extended training schedule [9]. In the present study, two training trials were given each day. The non-stressed controls displayed a seesaw-like pattern of performance, with longer latencies for the first trial of the day compared to the second trial of the previous day (long-term memory). The second trial of the day had short latencies, indicative for intact short-term working memory. Non-stressed mice displayed a smooth learning curve. However, stressed mice had a delay in learning but did improve their performance from day 3 onwards to the level of non-stressed mice. We regard the extended time in the start area, the slow walking and short distance walked during learning, expressions of behavioral inhibition in stressed mice, as it is also expressed during novelty exposure i.e. the first free exploration trial.

Post-training administration of sugar improved the performance of non-stressed controls. From day 2 onwards, latencies to the exit hole decreased from trial to trial (smooth learning curve), while controls without sugar were slower during the first trials of the training trials, resulting in a kind of “seesaw” pattern of performance. Treating the stressed mice with sugar revealed an interesting “normalization” of behavior. These mice displayed the same seesaw pattern of performance as non-stressed controls without sugar. However, this was a partial similarity to the behavior of controls as stressed mice with sugar had longer latencies during all first training trials of the day, and non-stressed controls improved over days. Nonetheless, post-training access to sugar could alleviate the effects of chronic stress and partially “normalize” the performance to the level of non-stressed mice. We consider this effect to be additional support for a chronic stress-induced alteration of the reward system. Concluding, the rewarding effects of sugar on memory depend on the prior life history, having experienced chronic stress or not.

In addition to a series of training trials over days, we challenged the mice with two conditions that require behavioral flexibility, changing behavior and learning strategies: (1) the exit hole is not available any more during the free exploration trials after spatial acquisition training; (2) the location of the exit hole was changed, i.e., reversal trials. The free exploration trials revealed that stressed mice use a more perservative strategy and are less flexible (returned more often to the same hole, remained longer in the area of the exit hole), as opposed to the more efficient serial strategy employed by the non-stressed mice. Focusing on the aspect of learning strategies, we recently reported that our chronic stress paradigm produces a shift in the use of search strategies by favoring stimulus-response over spatial learning strategies in mice and man [10]. Others [28] demonstrated in rats that chronic social stress caused a reorganization of the frontostriatal neuronal network and led to a bias of behavioral strategies towards habit (i.e., stimulus-response) learning. Acquiring the novel location of the exit hole is achieved by all mice. The free exploration trial following reversal training revealed that stressed mice returned to the original exit hole just as often as they returned to the new one, while non-stressed mice favored the new exit location. We might conclude that reversal learning is superior in the non-stressed mice. Surprisingly, latencies to exit were prolonged in non-stressed mice with sugar during reversal learning. Speculating, it might be that the original memory trace of the non-stressed mice with sugar is stronger than in the non-stressed without sugar, and therefore, interferes with the acquisition of new memory. For the stressed mice, post-training sugar has no apparent effect on reversal learning expressed by latencies to the new exit hole. The free exploration trial revealed behaviors of stressed mice with sugar that indicated increased flexibility, such as less perseveration and early approach of the rim area.

Emotions affect memory. It might be argued that changes in emotions, such as increased anxiety, contribute to the altered performance of the stressed mice. Behaviors related to anxiety and reduced risk-taking e.g., reduced speed of movements, reduced exploration and not visiting the rim area of the circular hole board, would support such a notion. In contrast, elevated anxiety is not expressed by stressed mice which remain long in the lit area of the light-dark box. Therefore, we prefer to consider a change in the behavioral inhibition, the balance between approach and avoidance as an acceptable operationalisation of behavior.

### Sucrose Consumption and Preference

The most common procedure to determine whether anhedonia has been induced in animals is the measurement of sucrose consumption and/or preference. Chronic stress most often decreases sucrose consumption when tested during and in close context with the applied stressor [30], [31]. In our previous study, chronic stress reduced sucrose consumption during the stress period and delayed the development of sucrose preference measured one day after the last stressor [7]. We can interpret this result as stress-induced anhedonia. In the present study we measured sucrose consumption 35 days after cessation of the stressor. Stressed mice consumed more volume of both sucrose and water. In contrast with our previous study, the sucrose consumption was not an indicator for anhedonia. Stressed mice even drink more fluid than non-stressed mice with the same preference for sucrose (88%) over water. In fact, we find a stress-induced increase of caloric intake. It is known that glucocorticoids stimulate behaviors that are mediated by the dopaminergic mesolimbic “reward” pathways, and increase the intake of food with high carbohydrate and fat [17], so-called “comfort” food, which contributes to the development of obesity.

Remarkably, and at this time unexplainable, is the finding that stressed mice that had received sugar during spatial training weeks before, preferred to drink water at the location where they had drunk sucrose the day before. Did they perceive the taste of sugar as highly rewarding, strengthening the memory for this location? It would be of great interest to study the time-dependent effects of chronic stress with respect to stress-induced metabolic changes and food intake.

Concluding, Chronic stress has immediate and long-lasting consequences for behavior, emotional and cognitive abilities. Especially the behavioral inhibition seems to become part of the daily repertoire of responses elicited by novelty, as well as in the familiar environment of the homecage. Corticosterone secretion patterns change, manifested as higher corticosterone levels during the day, within a week after cessation of the chronic stress procedure. Post-training reward in close context with a spatial learning task could partially rescue the chronic stress-induced behavioral changes that reflect emotions and cognitive processes.

We conclude that our chronic stress model results in behavioral and neuroendocrine features that might contribute to the development of stress-related psychopathologies, such as depression and anxiety disorders. This is supported by the expression of anhedonia in our model. Introducing context-related periods of reward, as we did in relation to spatial memory formation, can ameliorate some of the chronic stress effects. Several parameters of behavior became comparable between stressed and non-stressed control mice. Other features, such as the stress-induced increased consumption of sucrose and water were not counteracted. Sugar as a reward even strengthened the memory for the location of the sucrose. This could indicate a possibility for craving and thereby affecting consumption of high caloric nutrients in the future. Our study has provided some insight into the complex interaction of reward and stress. While there are clear positive consequences on memory formation, metabolic effects in relation to chronic stress need more attention in future studies.

## Materials and Methods

### Ethics Statement

Experiments were approved by the Local Committee for Animal Health, Ethics and Research of the University of Leiden. Animal care was conducted in accordance with the European Council Directive of 24 November 1986 (86/609/EEC).

### Animals

Male C57BL/6J mice (*n* = 40, 10 weeks old) were purchased from Janvier (France). Upon arrival at the animal facilities (Gorlaeus laboratory, Leiden/Amsterdam, Center for Drug Research, University of Leiden, The Netherlands), mice were transported to the experimental room to acclimatize for two weeks before the start of the experiment (days 1–14). They were housed individually in a temperature (21±1°C) and humidity (55±5%) controlled room, with food and water *ad libitum*; 12∶12 h light-dark cycle (lights on at 07∶00 am). Behavioral testing was performed between 09∶00 a.m. and 14∶00 p.m.

### Experimental Design

**Figure 5:**
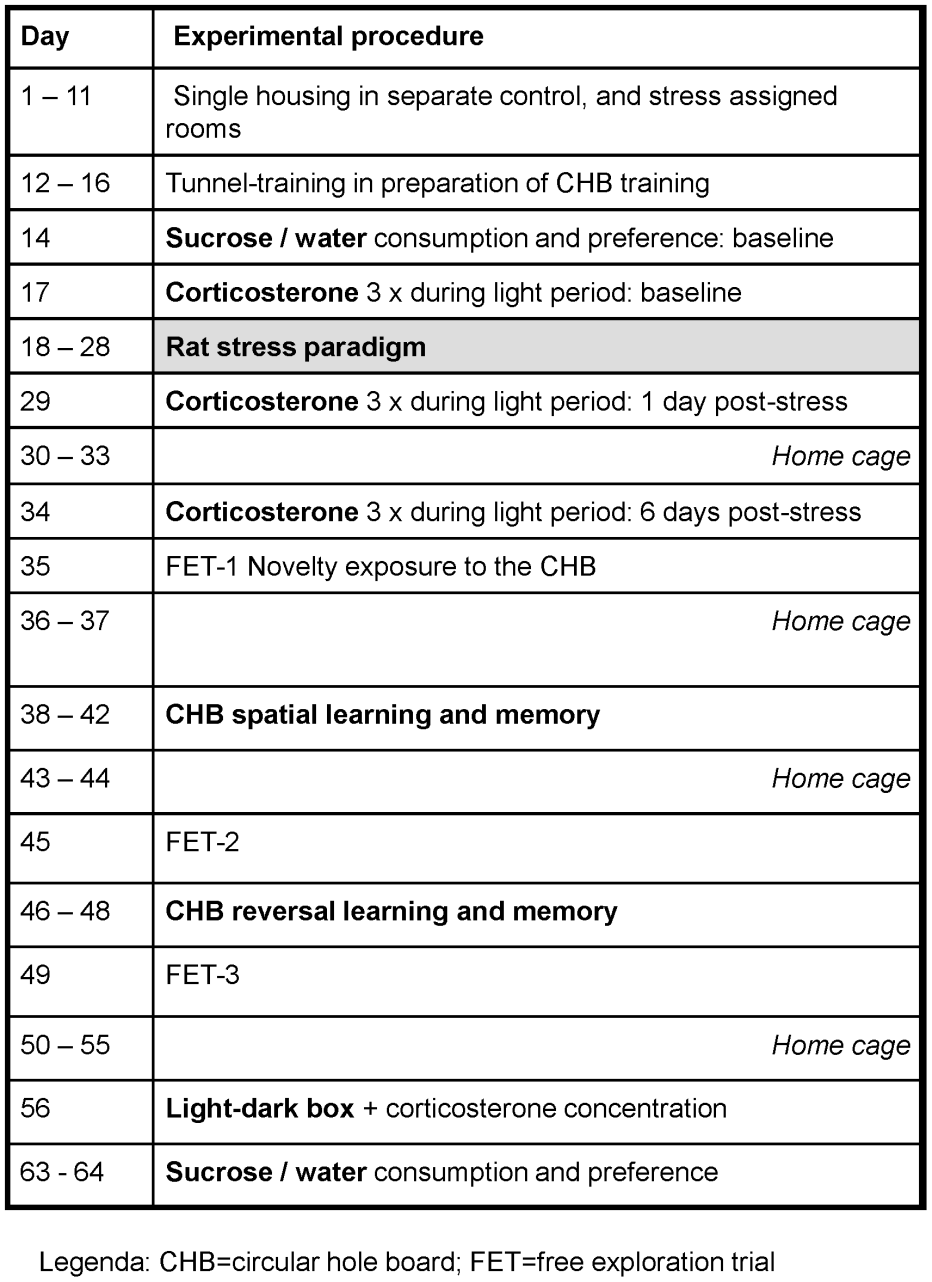
Corticosterone concentrations determined in blood plasma, before, and 1 and 6 days after chronic stress. Using tail-incision, blood was withdrawn during the light period of the day at A) 09∶00 a.m., 13∶00 and 17∶00 p.m. to determine the corticosterone (ng/ml) levels; B) Overall corticosterone concentration during the light period, expressed as Area Under the Curve (AUC_total). Data represent mean ± SEM; *p<0.05 1-day post-stress vs. before stress and/or 6-days post-stress.

Figure 5 depicts the timeline of the experiment. Mice were subjected to two conditions (*n* = 20/group; days 18–28); (i) stress: exposure to rats within a 2 week period and (ii) control: remaining undisturbed in the home cage. Endocrine (corticosterone), emotional and cognitive responses were assessed several times throughout the duration of the experiment. The corticosterone concentration was determined three times during the light period: baseline (day 17), and one- and six days after the last rat exposure (days 29 and 34). On day 35, mice were exposed for 5 min to the novel environment of the circular hole board (CHB). The CHB was subsequently used to test acquisition of spatial learning (days 38–42) and reversal learning (days 46–48). Exploration strategies were assessed on days 35, 45 and 49, i.e., before, after spatial-, and after reversal learning. Four weeks after cessation of the stressor (day 56), the behavioral response to the light-dark box environment was assessed. Immediately thereafter a blood sample was taken to determine the novelty-induced corticosterone concentration. A sucrose solution was available for 24 h before (day 15), and after ‘rat stress’ (day 63). Bodyweight was measured daily from the day of arrival until the end of the experiment.

Behavior was recorded on videotape and analyzed using EthoVision Windows 3.1 (Noldus Information and Technology BV, Wageningen, The Netherlands). The image analysis system sampled the position of the mouse 12.5 samples/second. To calculate the distance moved, we set the system to score movement when the mouse moved at least with a velocity of 3.5 cm/second, averaged over 12 samples.

### Rat Stress Paradigm

Exposure to a rat profoundly activates the Hypothalamic-Pituitary-Adrenal (HPA) axis of the mouse, resulting in elevated corticosterone concentrations in brain lysate [32] and blood plasma [9]. During the first week (days 18–22) of the ‘rat stress’ paradigm, mice were exposed to rats on 5 consecutive days: one or two hours a day, either morning or afternoon, resulting in a total rat-exposure time of 9 h. In the second week (days 26–28), two exposures took place: on Tuesday (1 h) and Thursday (1 h; see also [7].

One rat was placed on top of two mouse cages. Mice and rats were separated by a grid and could see, hear and smell, but not touch each other. Food and water were not available during rat exposure. To reduce predictability of the procedure for the mice, exposures took place at different times during the light phase. Furthermore, the location of the rat and the mouse cages were changed *ad random* within the experimental room. To avoid exposure to the smell of rats, the person who performed the rat stress procedure did not enter the separate housing room of the control mice. Control mice remained in their home cage. To assess the effect of rat exposure on arousal, mice were weighed before and directly after the last rat exposure of the day. Comparable time points were used for weighing the control mice.

### Blood Sampling and Corticosterone Measurement

To characterize the effect of the ‘rat stress’ paradigm at the endocrine level, we used the following procedure: The day before the start of the stress paradigm, and 1 and 6 days after the last rat exposure, a small blood sample was collected from the mice via tail-incision three times during the light period at 09∶00 a.m., 13∶00 and 17∶00 p.m. Briefly, a small incision at the base of the tail with a razor blade allows collection of a <50 µl blood, within 90 sec after opening of the animal’s cage [33]. Corticosterone was measured using a commercial ^125^I-corticosterone radioimmunoassay kit (MP Biomedicals, NY, USA; the intra-assay variability is 7.3%).

### Circular Hole Board

The apparatus is a grey round plate (PVC; diameter = 110 cm) with 12 holes (diameter = 5 cm) at equal distances from each other and at a distance of 10 cm from the rim of the hole to the rim of the plate, situated 1 m above the floor. Light intensity on the board surface was 120 lux. All holes could be closed by a lid at a depth of 5 cm. During learning trials one hole was open and connected to the home cage of the mouse by an s-shaped-tunnel (diameter = 5 cm×15 cm long). Only in close proximity to the hole (head into the hole) the mouse could see if it was open. Turning the board between trials, cleaning the surface before each mouse was placed on the board, and placing the home cage underneath the opposite exit hole during the free exploration trials, served to control odor cues (see for detailed description of the CHB apparatus and procedure [7].

Before a trial commenced the board was cleaned with 1% HAc, followed by turning the board clock- or anticlockwise until a randomly determined open hole was at the fixed location of the exit. The location of the exit hole changed between spatial acquisition- and reversal learning. The home cage of the mouse was placed underneath the board and was connected to the exit hole with an s-shaped-tunnel; the home cage was invisible to the mouse on the board. A trial started by placing the mouse in a grey cylinder (PVC, diameter = 10 cm; high = 25 cm) at the center of the board. After 10 sec the cylinder was lifted and the mouse could explore the CHB.

Mice were ‘pre-trained’ three times to climb through the s-shaped-tunnel during the week preceding the ‘rat stress’ paradigm (days 12–16). All mice readily entered and climbed through the tunnel at the third time of ‘pre-training’.

### Schedule and Procedure

Mice were run on the CHB between days 35–49. During free exploration trials (FET) all holes were closed by a lid; trials lasted 5 min: FET1: day 35 - novelty exposure; FET2: day 45 – three days after spatial acquisition training; FET3: day 49 – one day after reversal learning. Training trials were divided in (i) spatial acquisition (days 38–42): learning the location of an exit hole; (ii) reversal: learning the location of a new exit hole (days 46–48). A trial lasted 120 sec max, and two trials were run per day with an inter-trial-interval of 15 min. If the mouse did not locate the exit hole, it was gently guided towards the exit hole using a grid (20 cm×6 cm). A sub-group of the control and stressed mice received post-training 30 mg sugar, upon arrival in their home cage (in total 4 conditions, *n* = 10 mice/condition).

Overall, mice performed 16 learning trials (10 spatial acquisition and 6 reversals) and 3 FET’s. The following parameters were analyzed for the FET’s (i) general activity: path length (m), velocity (cm/s), number of holes visited; (ii) search strategies: sequence of hole visits (serial: more than two holes in sequence; perseveration: repeatedly visiting the same hole or alternately visiting two neighbouring holes), latency (s) and path length (m) to the exit holes as learned during spatial acquisition and reversal, number of visits to the exit holes, time spent in the zones (s) comprising of the hole adjacent left and right from the exit hole used during spatial- and reversal learning; (iii) anxiety related: latency (s) to leave the start center, latency (s) to the rim zone, number of rim dips, and number of boli. Training trials were analyzed for: latency (s) to leave the start center, latency (s) and path length (m) to exit hole, velocity (cm/s).

### Sugar Administration

On the first day of single housing a feeding cup (2.5 cm×2.3 cm) was taped to the bottom of the home cage in the corner opposite the nest [34]. All mice were familiarized with sugar on days 12 and 16 (i.e., before rat stress and CHB training commenced). The grid of the cage was lifted, the sawdust was removed from the feeding cup, and the sugar (30 mg) was added at 09∶00 a.m. Mice ate all the sugar within 15 min.

During the second spatial- and reversal training trials of the day, mice had free access to 30 mg sugar. All mice ate the sugar within 15 min after the trial, thus, in close context with the learning trial [14].

### Light-dark Box

On day 56 we determined the behavioral response of the mice to placement in the light compartment of the light-dark box and 5 min later blood samples were taken for the measurement of the corticosterone concentration. The plexiglass box was divided into a light- (30 cm×20 cm×25 cm; lux = 480) and darker compartment (15 cm×20 cm×25 cm; lux = 120). To start, mice were put in a grey cylinder (PVC, diameter = 10 cm; height = 25 cm), which was always placed in the same corner of the light compartment. After 10 sec the cylinder was lifted and the mouse was left to explore for 5 min. Thereafter, the box was swept clean with 1% HAc.

As behavioral parameters the time spent (%) and distance moved (cm) in the light compartment were assessed, as well as the latency (s) to enter the dark compartment and re-entry (s) into the light compartment.

### Sucrose Consumption and Preference

During sucrose testing mice had access to two bottles in their home cage, containing either water or a 5% sucrose solution. The first measurement of water and sucrose consumption, and preference was determined from day 14 to day 15: bottles were weighed before (day 14 at 09∶00 a.m.) and after 24 h (day 15 at 09∶00 a.m.). The reduction in weight of the bottles reflected the fluid consumption in ml; the difference in ml drunk from the water vs. the sucrose solution was calculated as percentage and reflects preference. These were taken as baseline values. The second sucrose testing was performed from day 63 to day 64 (between 09∶00 a.m. –09∶00 a.m.), which is 45 days after the last rat exposure.

After both sucrose testing days, the bottle containing the 5% sucrose solution was replaced by a water bottle. To assess whether sucrose consumption would affect the preference to drink water from a bottle placed at the location of the previously sucrose-containing bottle, water consumption was measured following the second sucrose test, for 24 h from day 64 to day 65.

### Statistical Analysis

Data were subjected to analysis of variance (ANOVA) with group of mice (controls and stress) and treatment (no-sugar, sugar), when appropriate with repeated measures, followed by a post-hoc LSD test (SPSS 15.0). Significance was accepted at p<0.05. Results are presented as mean ± standard error of the mean (SEM).

## References

[1] De Kloet ER, Vreugdenhil E, Oitzl MS, Joels M (1998). Brain corticosteroid receptor balance in health and disease.. Endocr Rev.

[2] McEwen BS (2005). Glucocorticoids, depression, and mood disorders: structural remodeling in the brain.. Metabolism.

[3] de Kloet ER, Joels M, Holsboer F (2005). Stress and the brain: from adaptation to disease.. Nat Rev Neurosci.

[4] Leppanen JM (2006). Emotional information processing in mood disorders: a review of behavioral and neuroimaging findings.. Curr Opin Psychiatry.

[5] Bevins RA, Besheer J (2005). Novelty reward as a measure of anhedonia.. Neurosci Biobehav Rev.

[6] DSM-IV-TR (2000). Diagnostic and Statistical Manual of Mental Disorders.. Washington DC: American Psychiatric Association Press.

[7] Dalm S, de Visser L, Spruijt BM, Oitzl MS (2009). Repeated rat exposure inhibits the circadian activity patterns of C57BL/6J mice in the home cage.. Behav Brain Res.

[8] Grootendorst J, de Kloet ER, Dalm S, Oitzl MS (2001). Reversal of cognitive deficit of apolipoprotein E knockout mice after repeated exposure to a common environmental experience.. Neuroscience.

[9] Grootendorst J, de Kloet ER, Vossen C, Dalm S, Oitzl MS (2001). Repeated exposure to rats has persistent genotype-dependent effects on learning and locomotor activity of apolipoprotein E knockout and C57Bl/6 mice.. Behav Brain Res.

[10] Schwabe L, Dalm S, Schachinger H, Oitzl MS (2008). Chronic stress modulates the use of spatial and stimulus-response learning strategies in mice and man.. Neurobiol Learn Mem.

[11] Huston JP, Mondadori C (1977). Reinforcement and memory: a model [proceedings].. Act Nerv Super (Praha).

[12] Huston JP, Oitzl MS (1989). The relationship between reinforcement and memory: parallels in the rewarding and mnemonic effects of the neuropeptide substance P. Neurosci Biobehav Rev.

[13] Messier C (2004). Glucose improvement of memory: a review.. Eur J Pharmacol.

[14] Dalm S, Schwabe L, Schachinger H, Oitzl MS (2009). Post-training self administration of sugar facilitates cognitive performance of male C57BL/6J mice in two spatial learning tasks.. Behav Brain Res.

[15] Strekalova T, Spanagel R, Bartsch D, Henn FA, Gass P (2004). Stress-induced anhedonia in mice is associated with deficits in forced swimming and exploration.. Neuropsychopharmacology.

[16] Willner P (2005). Chronic mild stress (CMS) revisited: consistency and behavioural-neurobiological concordance in the effects of CMS.. Neuropsychobiology.

[17] Dallman MF, Warne JP, Foster MT, Pecoraro NC (2007). Glucocorticoids and insulin both modulate caloric intake through actions on the brain.. J Physiol.

[18] Dallman MF (2007). Modulation of stress responses: how we cope with excess glucocorticoids.. Exp Neurol.

[19] File SE (2001). Factors controlling measures of anxiety and responses to novelty in the mouse.. Behav Brain Res.

[20] Kalueff AV, Keisala T, Minasyan A, Kuuslahti M, Tuohimaa P (2006). Temporal stability of novelty exploration in mice exposed to different open field tests.. Behav Processes.

[21] Powell SB, Geyer MA, Gallagher D, Paulus MP (2004). The balance between approach and avoidance behaviors in a novel object exploration paradigm in mice.. Behav Brain Res.

[22] Krebs RM, Schott BH, Schutze H, Duzel E (2009). The novelty exploration bonus and its attentional modulation.. Neuropsychologia.

[23] de Kloet ER, Oitzl MS, Joels M (1999). Stress and cognition: are corticosteroids good or bad guys?. Trends Neurosci.

[24] McEwen BS (1999). Stress and hippocampal plasticity.. Annu Rev Neurosci.

[25] Conrad CD (2010). A critical review of chronic stress effects on spatial learning and memory.. Prog Neuropsychopharmacol Biol Psychiatry.

[26] Roozendaal B, McEwen BS, Chattarji S (2009). Stress, memory and the amygdala.. Nat Rev Neurosci.

[27] Mizoguchi K, Yuzurihara M, Ishige A, Sasaki H, Chui DH (2000). Chronic stress induces impairment of spatial working memory because of prefrontal dopaminergic dysfunction.. J Neurosci.

[28] Dias-Ferreira E, Sousa JC, Melo I, Morgado P, Mesquita AR (2009). Chronic stress causes frontostriatal reorganization and affects decision-making.. Science.

[29] O’Carroll CM, Martin SJ, Sandin J, Frenguelli B, Morris RG (2006). Dopaminergic modulation of the persistence of one-trial hippocampus-dependent memory.. Learn Mem.

[30] Anisman H, Matheson K (2005). Stress, depression, and anhedonia: caveats concerning animal models.. Neurosci Biobehav Rev.

[31] Pothion S, Bizot JC, Trovero F, Belzung C (2004). Strain differences in sucrose preference and in the consequences of unpredictable chronic mild stress.. Behav Brain Res.

[32] Linthorst AC, Flachskamm C, Barden N, Holsboer F, Reul JM (2000). Glucocorticoid receptor impairment alters CNS responses to a psychological stressor: an in vivo microdialysis study in transgenic mice.. Eur J Neurosci.

[33] Dalm S, Enthoven L, Meijer OC, van der Mark MH, Karssen AM (2005). Age-related changes in hypothalamic-pituitary-adrenal axis activity of male C57BL/6J mice.. Neuroendocrinology.

[34] Dalm S, Brinks V, van der Mark MH, de Kloet ER, Oitzl MS (2008). Non-invasive stress-free application of glucocorticoid ligands in mice.. J Neurosci Methods.

